# Drug Reaction with Eosinophilia and Systemic Symptom (DRESS) induced by carbamazepine: a case report and literature review

**DOI:** 10.11604/pamj.2014.18.9.3799

**Published:** 2014-05-02

**Authors:** Nissrine EL Omairi, Sanae Abourazzak, Sanae Chaouki, Samir Atmani, Moustapha Hida

**Affiliations:** 1 Pediatric Department, Hassan II University Hospital, Fes, Morocco

**Keywords:** Epilepsy, carbamazepine, rash, DRESS syndrome

## Abstract

Drug-induced hypersensitivity or Drug Reaction with Eosinophilia and Systemic Symptom (DRESS) is a severe adverse drug-induced reaction. Diagnosing DRESS is challenging due to the diversity of cutaneous eruption and organs involved. Most of the aromatic anticonvulsants, such as phenytoin, phenobarbital, and carbamazepine, can induce DRESS. Culprit drug withdrawal and corticosteroids constituted the mainstay of DRESS treatment. We describe a 6 year-old boy who presented fever and rash 4 weeks after starting carbamazepine. Investigation revealed leukocytosis, atypical lymphocytosis, and elevated serum transaminases. The diagnosis of DREES syndrome was made, Carbamazepine was stopped and replaced initially by Clobazam and by Valproic acid after discharge, no systemic corticotherapy was prescribed. Symptoms began to resolve within two weeks, and by one month later her laboratory values had returned to normal. The aim of this work is to raise awareness general practitioner and pediatricians to suspect Dress syndrome in patients who present with unusual complaints and skin findings after starting any antiepileptic drug.

## Introduction

Drug rash with eosinophilia and systemic symptoms (DRESS) syndrome is a rather distinct severe adverse drug reaction. However, use of the term DRESS has been inconsistent, because eosinophilia is not a constant clinical finding, cutaneous and systemic signs are variable. The estimated incidence of this syndrome ranges from 1 in 1000 to 1 in 10,000 drug exposures, Adults are more affected than children [1]. The main culprit drugs are carbamazepine and allopurinol, even though 50 drugs can induce DRESS [2]. Another drug was reported particularly sulfa derivatives, antidepressants, non steroidal anti-inflammatory drugs, and antimicrobials. DRESS syndrome including a severe skin eruption, fever, hematologic abnormalities (eosinophilia or atypical lymphocytes), and internal organ involvement, usually 2-6 weeks after the initiation of drug therapy, with a possibility of persistence or aggravation of symptoms despite the discontinuation of the culprit drug. Given the potential morbidity, wide differential diagnosis, and relatively simple treatment, it is important for emergency physicians to consider this entity in patients with severe rashes. Severe cases of DRESS often require aggressive treatment; however, current pharmacologic treatment options are limited [2].

## Patient and observation

A 6 years boy was admitted in the emergency department for generalized pruritic erythematous oedematous patches, fever and chills. Our patient had partial epilepsy due to schizencephaly, not controlled by several antiepileptic drugs. Two months before the onset of symptoms, the patient began taking Carbamazepine, four weeks later he presented a rush especially in her abdomen 2 day later he became general and confluent, with facial and lip swelling, he complained of fatigue, headache, arthralgia, and fever (measured at 38, 7°), and aggravation in the frequency of seizure. Patient consults her general practitioner who prescribed antibiotics.

**Figure 1:**
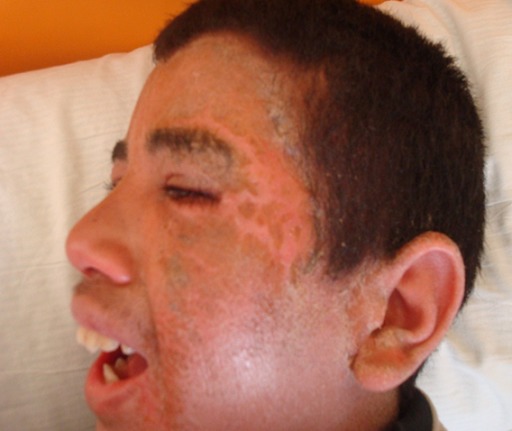
Erythematous and facial edema, with impetiginisation in the left side of the face

On admission patient's physical examination was remarkable for a fever 38, 5°, pulse rate was 134 beats per minute, blood pressure was 105/57 mm Hg, respiratory rate was 21 breaths/ min, and pulse-oximetry was 98% on room air. He was sleepy and discomfort. Her skin examination was significant for facial edema, lesion like impetigo in the left side of the face (Figure 1); he had also erythematous maculopapular eruption, most prominent on anterior chest, back, and extremities with keratinization of the palms and soles there were no bullae, and no mucous membrane or genital involvement. A mildly enlarged bilateral lymphadenopathy was palpated in her internal jugular chain. He exhibited no meningismus and had a normal heart, lung, and abdominal examination. Laboratory examination showed leukocytosis, white blood cell count 15.554/mm^3^ (normal range 4.000-10.000/ mm^3^), lymphocytosis 5678/ mm^3^, (normal range 1000-4000/mm^3^), (10% consisted of atypical lymphocytes with basophilic cytoplasm including vacuoles and larger than normal), basophilia 397/mm^3^ (0-150/mm^3^),monocytosis 1100/mm^3^(100-1000/mm^3^). No eosinophilia was noted. He has also abnormal liver function tests, aspartate aminotransferase (AST) 84 U/L (10-35 U/L), alanine aminotransferase (ALT) 67 U/L (10-40 U/L), and an elevated lactate dehydrogenase level 1235U/L (270-530 U/L). Her C-reactive protein was 65 mg/L (< 10 mg/L). Blood culture and bacteriological tests were negative.

The most common differential diagnoses include Stevens-Johnson syndrome (SJS), toxic epidermal necrolysis (TEN), Kawasaki disease and Still's disease. The patient was admitted to the pediatric department, to monitor for the development of worsening organ dysfunction, or toxic epidermal necrolysis. Carbamazepine was immediately stopped, and the patient was treated by oral antihistamines and dermal corticosteroids. For control of epilepsia attacks patient received Clobazam. Pharmacovigilance center was informed about this case.

**Figure 2:**
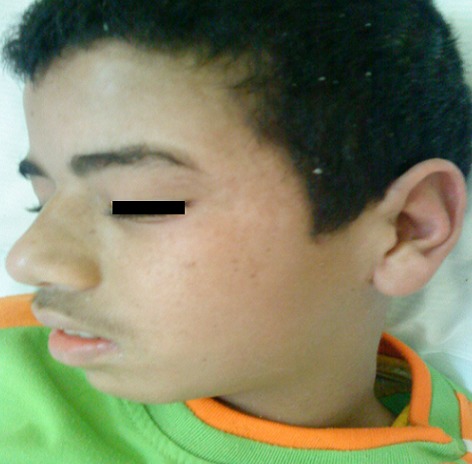
Patient two weeks after discharge

The patient's skin symptoms and laboratory abnormalities started improving on the tenth day of hospitalization. He returned at home after 2 weeks (Figure 2). One week after discharge, patient symptoms resolved. Her laboratory values returned to normal after one month. The patient epilepsy is now controlled by Valproic acid without any complication.

## Discussion

The acronym “drug rash with eosinophilia and systemic symptoms” was first introduced in 1996 by Bocquet, to describe patients exhibiting a drug-induced condition characterized by an extensive rash, fever, lymphadenopathy, hematologic abnormalities, hepatitis, and involvement of the kidneys, lungs, heart, or pancreas [3]. Usually presents within 8week. Aromatic anticonvulsants (phenytoin, phenobarbital, and carbamazepine) are the most common cause of DRESS. In the review of 172 cases reported as DRESS or drug hypersensitivity syndrome in the literature by using the Regis CAR scoring system [4], carbamazepine remains the mostly reported (27% of cases). But a variety of other drugs, such as allopurinol, minocycline, dapsone, sulfasalazine, and mexiletine, antiretrovirals have also been associated with DRESS. Cross-sensitivity is as high as 75% among the aromatic anticonvulsants [5].

The pathogenesis of DRESS syndrome is partially understood. Different mechanisms have been implicated in its development, including detoxification defects leading to reactive metabolite formation and subsequent immunological reactions slow acetylation, and reactivation of human herpes, including Epstein-Barr virus and human herpesvirus (HHV)-6 and -7. The detection of HHV-6 reactivation has even been recently proposed as a diagnostic marker for DRESS. Other types of viral infection were reported, such as cytomegalovirus reactivation and paramyxovirus infection. It is increasingly apparent that there is a genetic predisposition to adverse drug reactions. Studies have found that HLA-B*1502 is associated with carbamazepine induced Stevens-Johnson syndrome /toxic epidermal necrolysis in some Asian populations, but the same association does not occur in hypersensitivity syndrome (HSS) / DRESS. It is hoped that further research may define pharmacogenetic disease susceptibility markers to identify people at high risk of developing HSS / DRESS [6, 7].

Regis CAR and Japanese consensus group have developed specific criteria for making the diagnosis of DRESS. To meet the definition of DRESS, patients must have three of the four main Regis CAR criteria: an acute rash, fever above 38° C, lymphadenopathy at two sites, involvement of at least one internal organ, and abnormalities in lymphocyte and eosinophil counts. Additional criteria include hospitalization and that the reaction is suspected to be drug-related. Concerning Japanese consensus group the diagnosis requires meeting seven of the nine criteria in this system or all of the first five: a maculopapular rash developing > 3 weeks after drug initiation, clinical symptoms continuing > 2 weeks after stopping therapy, fever > 38° C, liver abnormalities (ALT > 100 IU/L) or other organ involvement, leukocytosis, atypical lymphocytes, eosinophilia, lymphadenopathy, or HHV-6 reactivation. Although an association with HHV6 is of interest, definite confirmation that herpes viruses are central to the hypersensitivity reaction is currently lacking. An ambiguous role for virus reactivation coupled with low availability of the relevant assay reduces the worth of HHV6 as diagnostic criteria [8].

The patient described here met the majority of criteria according to Regis CAR scoring guidelines for a diagnosis of DRESS induced by carbamazepine. Our patient didn't have eosinophilia but other hematological disorders like atypical lymphocytosis and monocytosis.

There is a 10% mortality rate from DRESS, mostly due to liver damage thought to be secondary to eosinophilic infiltration. The most important steps in managing patients with DRESS are recognizing the presence of this syndrome and immediately stopping the offending drug. Systemic corticosteroids have been considered the treatment of choice, especially in patients with internal organ involvement. The French Society of Dermatology published a report in 2010 outlining a consensus on therapeutic management of DRESS [9]. They recommend the use of systemic corticosteroids at a dose equivalent to 1 mg/ kg/day of prednisone in patients with any sign of severity including: transaminases greater than five times normal, renal involvement, pneumonia, hemophagocytosis, or cardiac involvement. They further recommend the use of IVIG at a dose of 2 g/kg over five days for a patient with life-threatening signs such as renal failure or respiratory failure. In addition, they propose the use of steroids in combination with ganciclovir in patients with signs of severity and confirmation of a major viral reactivation of HHV-6 [9].

N-acetylcysteine (NAC) has been proposed as a potential therapy because of its role in drug detoxification. Clinical and biochemical improvement has been obtained after the introduction of high doses of N-acetylcysteine in case reports of DRESS syndrome due to anticonvulsants [10]. Moreover, an unexpected side-effect of this therapy was the development of angio-oedema of the face. Further study and randomized controlled trials of these and other potential pharmacologic therapies will be important in establishing a standard of care and to improve understanding of how best to treat patients affected by DRESS syndrome.

## Conclusion

Although the mechanisms underlying DRESS syndrome remain poorly understood. This is a growing case reported in literature. The diagnosis of dress should be highly suspected with the presence of skin rash, liver involvement, fever, hypereosinophilia, and lymphadenopathy particularly in starting any new anti-epileptic. The use of systemic corticosteroids is classically reported in case of organ- or life-threatening disease. However, this remains controversial and may lead to activation of HHV- 6. Besides the prompt withdrawal of causative drug as standard of care, further studies are needed to recommend specific treatment guidelines.
